# The relationship between self-esteem and aggressive behavior among Chinese adolescents: A moderated chain mediation model

**DOI:** 10.3389/fpsyg.2023.1191134

**Published:** 2023-06-12

**Authors:** Yuanyan Hu, Yi Cai, Rui Wang, Yihan Gan, Nianqin He

**Affiliations:** ^1^Laboratory of Emotion and Mental Health, Chongqing University of Arts and Sciences, Chongqing, China; ^2^Postdoctoral Workstation of Art Theory, Southeast University, Nanjing, China; ^3^School of Education, Chongqing University of Arts and Sciences, Chongqing, China; ^4^Teacher Education Center, Anshan Normal University, Anshan, China; ^5^Department of Psychology, Boston University, Boston, MA, United States; ^6^Southwest Hospital Affiliated to Army Military Medical University, Chongqing, China

**Keywords:** adolescents, self-esteem, jealousy, self-control, aggressive behavior, gender

## Abstract

This study aimed to examine the relationship between adolescent self-esteem and aggressive behavior. Specifically, a moderated chain mediation model was developed to investigate the mediating role of jealousy and self-control and the moderating role of gender. Data were collected from 652 Chinese adolescents who completed the Self-Esteem Scale, Self-Report Jealousy Scale, Self-Control Scale and Aggressive Behavior Questionnaire. Results showed that adolescent self-esteem may significantly negatively affect aggressive behavior by mediating with jealousy and self-control. Moreover, gender possibly moderates the serial mediating effect of jealousy and self-control between adolescent self-esteem and aggressive behavior. The results have important theoretical and practical implications in that these reveal the influencing factors of adolescent aggressive behavior and the pathways to reduce such behavior.

## 1. Introduction

China has the second largest number of adolescents in the world, and about 51% of adolescents show high levels of aggression in secondary school (Hamza et al., 2019; Ning, 2021). Aggressive behavior refers to behavior that is intentionally directed towards others and has a negative impact on them, either physically or psychologically (Anderson and Bushman, 2001). The higher the level of aggression, the more likely to develop criminal behavior, which is not conducive to social harmony and stability (Li et al., 2019). In addition, aggressive behavior can negatively affect many aspects of adolescents, such as damaging the physical and mental health of the person attacked (Card et al., 2008) and resulting in poorer peer relationships and lower grades for the attacker (Ren et al., 2021). Thus, this study attempts to investigate the factors that influence adolescent aggressive behavior and the mechanisms behind its formation and provide an empirical basis and theoretical guidance for reducing adolescent aggressive behavior.

Individuals will have physiological changes such as increased secretion of sex hormones in adolescence, and psychological characteristics such as fluctuations in self-esteem levels, diverse emotional changes, and weakened self-control, and various sudden contradictions increase practical characteristics, which will increase adolescent aggressive behavior (González et al., 2017). Self-esteem is a positive feeling and an evaluation of self-worth that people develop during the process of socialization, which affects the development of individual behavior (Pan, 2015). Previous studies have also shown that self-esteem plays an important role in adolescent aggressive behavior, and aggressive behavior can be predicted well (Hu, 2009; Turner and White, 2015). Specifically, adolescents with low self-esteem are prone to receive negative feedback from the external environment and to become insensitive to positive feedback, which makes them susceptible to anger, jealousy, and dysfunctional behavior such as uncivilized and aggressive behavior (Hu, 2009). Thus, we propose hypothesis H_1_: Adolescent self-esteem negatively predicts aggressive behavior.

Jealousy is a negative emotional experience consisting of anger and resentment when comparing oneself to others and finding oneself inferior to them in some way (Lin et al., 2003). According to emotion regulation theory, the emotional experience of jealousy can influence adolescent aggressive behavior and can significantly predict individual problem behavior (Thompson, 1991; Murphy and Russell, 2018; Gao et al., 2022). Compared with individuals in other age groups, adolescents experience jealous emotions more often (Lennarz et al., 2017) and this feeling is often accompanied by aggressive behavior (Murphy and Russell, 2018). Studies have found that self-esteem can significantly negatively predict jealousy (Broemer and Diehl, 2003; Goldenberg et al., 2003; Si, 2009). It may be that most individuals with low self-esteem have lower levels of mental development than individuals with high self-esteem. Even if they are temporarily inferior to others, individuals with low self-esteem do not believe that they can catch up with or even surpass others through hard work, so individuals with low self-esteem are prone to jealousy (He and Zhang, 2012). Furthermore, the self-hierarchical model suggests that self-esteem, as a higher-level psychological trait in the model, affects the perception of self and others, which in turn affects individuals’ emotional experiences (Yang et al., 2017), such that those with low self-esteem have stronger emotional reactions to negative self-related information and are more likely to experience more jealousy when comparing themselves to others (Zhang et al., 2006; Chin et al., 2016), which ultimately leads to increased aggressive behavior. A study has also found that jealousy mediates between adolescent self-esteem and aggressive behavior (Sun et al., 2013). Thus, we propose hypothesis H_2_: Jealousy plays a mediating role between self-esteem and aggressive behavior in adolescents.

Self-control refers to the replacement of an individual inherent behavioral responses with other behavior in order to overcome their own desires and needs, and thus enable them to adapt to society more effectively (Baumeister and Tice, 2007). It is one of the factors affecting adolescent aggressive behavior and can effectively predict aggressive behavior (Denson et al., 2012). Specifically, improved self-control can reduce the incidence of aggressive behavior and even criminal behavior (Pechorro et al., 2021). Previous studies have shown that self-control can be influenced by self-esteem (Finkenauer et al., 2005), such that individuals with low self-esteem are more likely to lose self-control and adopt negative coping styles such as aggressive behavior when faced with an external stimulus (Li et al., 2017). The general theory of crime also suggests that the influence of all psychological traits on problem behavior must be achieved through self-control (Barlow, 1991). Therefore, as a psychological trait, self-esteem may indirectly affect adolescent aggressive behavior through self-control. Furthermore, another study found that self-control mediates the role between self-esteem and aggressive behavior (Huang et al., 2013). Thus, we propose hypothesis H_3_: Self-control mediates the role between adolescent jealousy and aggressive behavior.

Studies have shown that jealousy as a negative emotional experience can directly affect an individual’s level of self-control, which in turn affects the generation of behavior. Based on the strength model of self-control, adolescents will deplete their limited available psychological resources in order to counteract and control their jealous reactions (Crusius and Mussweiler, 2012; Yu et al., 2013), which decreases their self-control and thus increases the production of aggressive behavior (Hill et al., 2011). In addition, previous studies have verified that self-esteem affects adolescent jealousy and increase in an individual self-esteem and sense of power can reduce the intensity of their emotional experience of jealousy (Chin et al., 2016). Thus, we propose hypothesis H_4_: Jealousy and self-control possibly play significant chain mediating effects between self-esteem and aggressive behavior. In addition, due to the influence of gender cognition, personality traits, living habits, there are gender differences between males and females in emotional experience (Chen, 2010). Previous study has shown significant differences in jealousy between males and females (Zhang et al., 2006). Thus, we propose hypothesis H_5_: Gender regulates the influence of self-esteem on jealousy.

**Figure 1:**
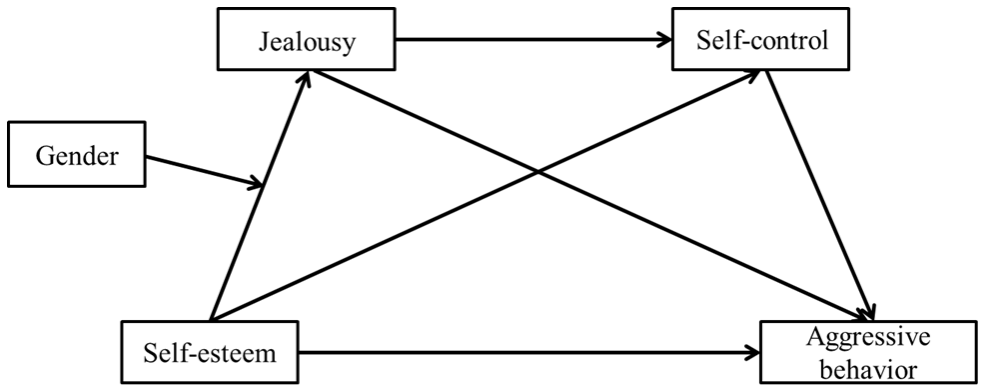
The proposed hypothesis model.

In summary, this study proposes a hypothesis model (Figure 1) and tests the above hypothesis through empirical research, which provides theoretical guidance and empirical basis for scientific prevention and effective control of the occurrence of adolescent aggressive behavior.

## 2. Methods

### 2.1. Participants

A total of 680 participants who not suffered from psychological, neurological, or major physiological diseases were selected from a secondary school in Chongqing, using the cluster sampling method. After excluding incomplete and inattentive questionnaires, 652 valid questionnaires were obtained. The effective ratio of the questionnaire is 95.9% and the mean age of the participants was 14.18 ± 1.25 years old (Table 1). Additionally, consent was obtained from all participants and relevant school personnel, before the study.

**Table 1:** Basic participant information.

	**Gender**		**Grade**			
	Male	Female	Junior 1	Junior 2	Junior 3	Senior 1
Number	335	317	170	174	153	155

### 2.2. Measures

#### 2.2.1. The self-esteem scale

Self-esteem was assessed with the Self-Esteem Scale (SES),which was developed by Rosenberg (Blascovich and Tomaka, 1991), modified by Tian (2006) and applied to Chinese participants. Responses on the 10 items (e.g., “I feel that I have a number of good qualities”) were rated on 4-point Likert scales ranging from 1 (strongly disagree) to 4 (strongly agree). A higher total score indicates a higher level of self-esteem of the individual. In this study, the internal consistency was *α* = 0.83.

#### 2.2.2. The self-report jealousy scale

Jealousy was assessed with the Self-report Jealousy Scale (SJS), which developed by Bringle and Boebinger (1990), and was revised by Wang (2001). Responses on the 20 items were rated on 3-point Likert scales ranging from 1 (not jealous) to 3 (strongly jealous). The higher the total score, the higher the level of jealousy of the individual. This measure has high reliability and validity and is applicable to Chinese participants (Wang, 2001). In this study, the internal consistency was *α* = 0.85.

#### 2.2.3. The self-control scale

Self-control was assessed by using the Self-control Scale (SCS) developed by Boone et al. (2004), which Tan and Guo (2008) modified for secondary school students, and this scale has good reliability and validity (Hu et al., 2012). Responses on the 19 items were rated on 5-point Likert scales ranging from 1 (strongly disagree) to 5 (strongly agree). The higher the total score, the lower the level of self-control exhibited by the individual. In this study, the internal consistency was *α* = 0.87.

#### 2.2.4. The aggression questionnaire

Aggressive behavior was assessed with the Aggression Questionnaire (AQ), developed by Buss et al. (1992) and revised by Liu et al. (2009). Responses on the 20 items were rated on 5-point Likert scales ranging from 1 (strongly disagree) to 5 (strongly agree). The higher the total score, the more aggressive the individual is. In this study, the internal consistency was *α* = 0.9.

### 2.3. Statistical processing

We used the Harman’s one-way method to test common method variance (CMV) and the SPSS macroPROCESS prepared by Hayes to test for the proposed hypothesis model.

## 3. Results

### 3.1. Common method variance

During the questionnaire survey, various methods were employed to reduce the occurrence of common method variance (CMV). These included the use of anonymity, guided participants to report truthfully, and the use of reverse scoring questions. In addition, the Harman one-way method was applied to test for CMV. The results showed that the variance explained by the first factor was 19.75%, which was below the critical value of 40% (Xiong et al., 2012), indicating that there was no serious CMV issue in this study.

### 3.2. Descriptive statistics and correlation analysis

According to correlation analysis, there were significant negative correlations between self-esteem, jealousy and aggressive behavior; a significant positive correlation between self-esteem and self-control; a significant negative correlation between jealousy and self-control; and a significant positive correlation with aggressive behavior. Moreover, a significant negative correlation was observed between self-control and aggressive behavior (Table 2).

**Table 2:** Descriptive statistics and correlation analysis of each variable (*n* = 652).

**Variable**	***M ± SD***	**1**	**2**	**3**	**4**	**5**
1. Gender	0.51 *±* 0.50	—				
2. Self-esteem	26.96 ± 4.65	0.12^**^	—			
3. Jealousy	40.02 *±* 6.97	−0.12^**^	−0.18^**^	—		
4. Self-control	54.92 *±* 11.72	0.04	0.56^**^	−0.35^**^	—	
5. Aggressive behavior	53.09 *±* 13.36	−0.10^**^	−0.42^**^	0.36^**^	−0.67^**^	—

** *p *< 0.01; gender(“0” = female, “1” = male).

**Table 3:** Test for variability of variables.

	**Self-esteem**	**Jealousy**	**Self-control**	**Aggressive behavior**
Gender				
Male	27.57 ± 4.80	39.08 ± 6.74	54.44 ± 12.36	54.64 ± 12.55
Female	26.31 ± 4.39	41.02 ± 7.08	55.44 ± 11.00	51.61 ± 13.95
*t*	−3.49^**^	3.58^***^	−1.09	2.92^**^

***p  < 0.001, **p < 0.01.

### 3.3. Test for variability of variables

Findings showed that Chinese adolescents have significant gender differences score of on self-esteem, jealousy and aggressive behavior (*p* < 0.01). Specifically, males scored higher than females on self-esteem, while females scored higher than males on jealousy and aggressive behavior. However, the gender difference was not significant in the scores of self-control (*p* > 0.05).

### 3.4. Regression analysis

The correlation analysis met the statistical requirements for testing the mediating effect of jealousy and self-control (Wen and Ye, 2014). The results, presented in Table 4, demonstrated a significant effect of both self-esteem and gender on jealousy (*β* = −0.16 ~ −0.12, *p* < 0.01); jealousy and jealousy effectively predicted self-control (*β* = −0.27 ~ 0.48, *p* < 0.001). Moreover, jealousy and self-control both effectively predicted aggressive behavior (*β* = −0.57 ~ 0.14, *p* < 0.01).

**Table 4:** Regression analysis of each variable.

**Variable**	**Predictor**	***R*** ^**2**^	***ΔR*** ^***2***^	***F***	***β***	***t***
Jealousy	Self-esteem	0.05	0.04	15.56^***^	−0.16	−4.23^***^
	Gender				−0.12	−3.03^**^
Self-control	Self-esteem	0.35	0.35	172.53^***^	0.48	−14.83^***^
	Jealousy				−0.27	−8.34^***^
Aggressive behavior	Self-esteem	0.47	0.47	193.19^***^	−0.10	−3.00^**^
	Jealousy				0.14	4.53^***^
	Self-control				−0.57	−16.06^***^

***p  < 0.001, **p < 0.01.

### 3.5. Mediation analyses

The results of the regression analysis met the statistical requirements for conducting a test of mediating effects (Wen and Ye, 2014). Thus, the hypothesis was subsequently tested using model 83 based on the bias-corrected percentile bootstrap method (5,000 replicate samples), the results of which are shown in Table 5. The direct effect of adolescent self-esteem on their aggressive behavior was found to be statistically significant (*β* = −0.22, *p* < 0.05). Furthermore, bootstrap 95% confidence intervals for the total mediating effect of jealousy and self-control were found to not include 0, thus indicating that jealousy and self-control play a significant chain mediating effect between self-esteem and aggressive behavior, accounting for 81.22% of the total effect.

**Table 5:** The test of mediating effects.

Path	Effect size	Boot standard error	Proportion of mediating effect	95% Confidence interval	
				Lower limit	Upper limit
Indirect effect 1	−0.07	0.02	5.54%	−0.12	−0.03
Indirect effect 2	−0.83	0.08	69.44%	−1.00	−0.67
Indirect effect 3	−0.07	0.02	6.24%	−0.12	−0.03
Total mediating effect	−0.97	0.09	81.22%	−1.40	−0.99
Direct effect	−0.22	0.10		−0.41	−0.04
Total effect	−1.20				

*Indirect effect 1, Self-esteem→Jealousy→Aggressive behavior; Indirect effect 2, Self-esteem→Self-control→Aggressive behavior; Indirect effect 3, Self-esteem→Jealousy→Self-control→Aggressive behavior*.

### 3.6. Regulation analysis

**Figure 2:**
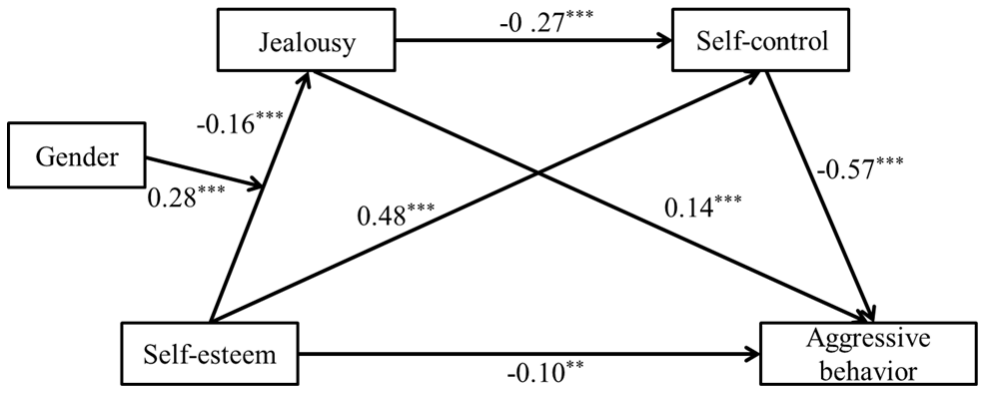
The relationship between adolescent self-esteem and aggressive behavior: the mediating role of jealousy and self-control and the moderating role of gender. ***p < 0.001, **p < 0.01.

**Figure 3:**
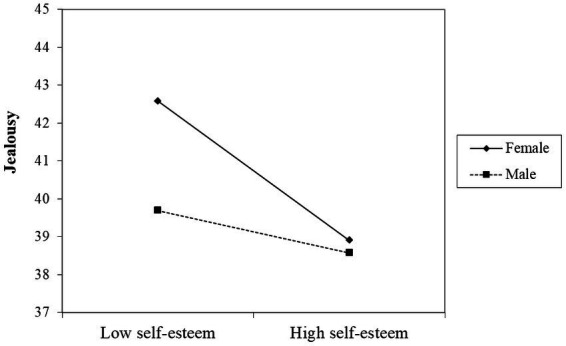
Figure of regulating effect.

Figure 2 demonstrates that the interaction between self-esteem and gender has a significant influence on jealousy (*β* = 0.28, *p* < 0.001) with the 95% confidence interval [0. 01, 0.16]. This indicates that gender significantly moderates the relationship between self-esteem and jealousy. To further elucidate the moderation effect of gender, a simple slope test was conducted based on the methodology proposed by Toothaker (1994), as illustrated in Figure 3. The results showed that lower levels of self-esteem predicted higher levels of jealousy in females (*β* = −0.40, *t* = −4.61, *p* < 0.001), while the result of males was not significant (*β* = −0.12, *t* = −1.53, *p* > 0.05).

## 4. Discussion

In recent years, the increasing prevalence of aggressive behavior among adolescents has become a major public health concern globally (Liu et al., 2016; Robert et al., 2017). This study revealed that adolescent self-esteem negatively predicted their aggressive behavior, which is consistent with the findings of previous studies (Brentdonnellan et al., 2005; Luo et al., 2011). This may be because adolescents with low self-esteem lack social connections, which makes them maladaptive to social norms and leads to an increase in aggressive behavior (Brentdonnellan et al., 2005). Therefore, it is essential to enhance the self-esteem of adolescents so that they can maintain physical and mental health and promote harmonious stability in society. Additionally, in order to further explore the mechanisms of adolescent aggressive behavior, this study used jealousy and self-control as mediating variables, and gender as a moderating variable, and some meaningful conclusions were obtained.

The results of this study indicate that jealousy mediates the relationship between adolescent self-esteem and aggressive behavior. Prior research has shown that adolescents with lower self-esteem are more prone to make negative social comparisons with others, leading to higher levels of jealousy (Desteno and Salovey, 1996; Zhang et al., 2006; Rentzsch et al., 2015). Moreover, adolescents pay more attention to negative external feedback, undergo more jealous experiences (Chin et al., 2016), and are less able to effectively manage their emotions, compared to other individuals, which can easily lead to more aggressive and even criminal behavior (Desteno et al., 2006; Gu et al., 2012; Liu et al., 2021). Therefore, adolescents with lower self-esteem may be more susceptible to negative information about their perceptions of self and others and may be more prone to jealousy in social comparisons, which may lead to the engagement of aggressive behavior as a negative coping style.

The results indicate that self-control mediates the relationship between self-esteem and aggressive behavior, suggesting that self-esteem can influence aggressive behavior through self-control. Previous studies have shown that adolescent self-esteem levels affect self-control, with a lack of social support reducing individual self-esteem levels and consequently leading to a lower self-efficacy and less self-control (Huang et al., 2013; Li et al., 2017). In addition, the strength model of self-control states that an individual ability to exercise self-control depends on their limited energy and continuous emotional regulation will gradually reduce the energy of self-control (Yu et al., 2013). Consequently, a lack of self-control energy can lead to problematic behavior such as aggressive behavior (Dewall et al., 2007). Thus, adolescents with low self-esteem may continuously use their limited self-control energy to help them adapt to society during socialization, which leaves them with fewer self-control resources and increases the likelihood of aggressive behavior.

Moreover, the results suggested that adolescent jealousy and self-control possibly played chain mediating effects in between self-esteem and aggressive behavior, which further explained the mechanism by which adolescent self-esteem influences aggressive behavior. According to the emotion regulation theory, adolescents with low self-esteem are more likely to feel inferior and jealous when comparing themselves to others, increasing their sensitivity to social events and leading to aggressive behavior as a way to conform to social norms (He et al., 2012; Cao and Zhang, 2018). At the same time, they tend to exhibit aggressive behavior in order to ensure their emotional responses conform to social norms and consume their self-control energy (Dewall et al., 2016). Additionally, the broaden-and-build theory of positive emotions proposes that such emotions can expand the cognitive and behavioral range of individuals and improve the utilization rate of their psychological energy (Fredrickson, 2004). Thus, appropriately improving the self-esteem level of adolescents may make them often have a positive attitude towards themselves and feel positive emotional experiences, rather than negative emotional experiences such as jealousy (Si, 2009). Furthermore, they can then fully use their self-control energy to actively control their cognition and behavior, thereby reducing the occurrence of aggressive behavior (Agbaria, 2021).

Finally, this study found that gender may have a moderating effect between self-esteem and jealousy. Specifically, females with low self-esteem were more likely to develop higher levels of jealousy than males. This could be because females and males have different thinking styles, cognitive styles, etc., particularly as girls who enter adolescence, they tend to have more delicate feelings and pay more attention to details (Li and Yang, 2008). Additionally, individuals with low self-esteem were more sensitive to negative information and perceive themselves as inferior to others (Chin et al., 2016). As a result, females with low self-esteem were more likely to experience jealousy in relationships, studies, life, and other areas of life.

### 4.1. Implications

Theoretically, we more comprehensively discuss the internal action path of the impact of adolescent self-esteem and aggressive behavior and deepen the understanding of the mediation mechanism of aggressive behavior, while providing theoretical guidance and empirical evidence for preventing and reducing such behavior. From a practical perspective, we found that it is possible to reduce the experience of jealousy and thus aggressive behavior by enhancing adolescent self-esteem levels. This can be achieved through greater care and support by family members, teachers and peers or friends. Moreover, the findings suggest that it is possible to improve self-control energy by improving emotion management ability and teaching adolescents how to face external negative information in a positive way, thus reducing the possibility of aggressive behavior. Additionally, special attention needs to be paid to female’s self-esteem levels and their ability to focus on positive information.

## 5. Limitations

This study has some limitations that need to be further improved. First, the participants were all middle school students in school, but there are many non-school adolescents in real life. Therefore, future studies on this topic could use a larger sample that includes the latter as well. Second, the use of a single questionnaire may have a social approval effect, and multiple methods can be used to study this issue in the future, such as implicit association tests and event-related potential techniques, which are less affected by social approval. Third, this study finds that self-control is playing a much larger role than jealousy. However, the reasons for this have not been further studied. Therefore, we could explore the reasons it in future studies. Fourthly, since this is a cross-sectional study, it cannot draw conclusions about causality from the data. Therefore, further investigation through longitudinal studies is necessary to understand the causal relationship between the variables.

## 6. Conclusion

This study established a regulatory chain mediation model. Specifically, adolescent self-esteem may significantly negatively affect aggressive behavior by mediating with jealousy and self-control. Moreover, gender possibly moderates the serial mediating effect of jealousy and self-control between adolescent self-esteem and aggressive behavior. This information would be of significant value to those professionals engaged in helping adolescents to address their behavioral issues.

## Data availability statement

The original contributions presented in the study are included in the article/Supplementary material, further inquiries can be directed to the corresponding author.

## Ethics statement

The studies involving human participants were reviewed and approved by All procedures performed in the study were in accordance with the ethical standards of the Research Ethical Committee in the Ethics Committee of Chongqing University of Arts and Sciences. Written informed consent to participate in this study was provided by the participants’ legal guardian/next of kin.

## Author contributions

YH, YC and NH contributed to the conception of the study and drafted the manuscript. YC collected the data and performed the statistical analysis. RW, YG and NH worked as writer’s assistants. YH, RW, YG and NH provide financial Support for this project. All authors contributed to the article and approved the submittedversion.

## Conflict of interest

The authors declare that the research was conducted in the absence of any commercial or financial relationships that could be construed as a potential conflict of interest.

## Publisher’s note

All claims expressed in this article are solely those of the authors and do not necessarily represent those of their affiliated organizations, or those of the publisher, the editors and the reviewers. Any product that may be evaluated in this article, or claim that may be made by its manufacturer, is not guaranteed or endorsed by the publisher.

## References

[ref1] Agbaria Q. (2021). Internet addiction and aggression: the mediating roles of self-control and positive affect. Int. J. Ment. Heal. Addict. 19, 1227–1242. doi: 10.1007/s11469-019-00220-z

[ref2] Anderson C. A. Bushman B. J. (2001). Effects of violent video games on aggressive behavior, aggressive cognition, aggressive affect, physiological arousal, and prosocial behavior: a meta-analytic review of the scientific literature. Psychol. Sci. 12, 353–359. doi: 10.1111/1467-9280.00366, PMID: 11554666

[ref3] Barlow H. D. (1991). A general theory of crime. Journal of Criminal Law and Criminology. 82, 229–242. doi: 10.2307/1964276

[ref4] Baumeister R. F. Tice V. (2007). The strength model of self-control. Curr. Dir. Psychol. Sci. 16, 351–355. doi: 10.1111/j.1467-8721.2007.00534.x

[ref5] Blascovich J. Tomaka J. (1991). Measures of self-esteem. Measures of Personality and Social Psychological Attitudes. 1:18. doi: 10.1016/B978-0-12-590241-0.50008-3

[ref6] Boone A. L. Tangney J. P. Baumeister R. F. (2004). High self-control predicts good adjustment, less pathology, better grades, and interpersonal success. J. Pers. 72, 271–324. doi: 10.1111/J.0022-3506.2004.00263.X, PMID: 15016066

[ref7] Brentdonnellan M. Trzesniewski K. H. Robins R. W. Moffitt T. E. Caspi M. A. (2005). Low self-esteem is related to aggression, antisocial behavior, and delinquency. Psychol. Sci. 16, 328–335. doi: 10.1111/j.0956-7976.2005.01535.x 15828981

[ref8] Bringle R. G. Boebinger K. L. G. (1990). Jealousy and the 3rd-person in the love triangle. J. Soc. Pers. Relat. 7, 119–133. doi: 10.1177/0265407590071007

[ref9] Broemer P. Diehl M. (2003). Romantic jealousy as a social comparison outcome: when similarity stings. J. Exp. Soc. Psychol. 40, 393–400. doi: 10.1016/j.jesp.2003.08.002

[ref10] Buss P. A. H. Mark (1992). The aggression questionnaire. J. Pers. Soc. Psychol. 63, 452–459. doi: 10.1037//0022-3514.63.3.452 1403624

[ref11] Cao X. Zhang L. (2018). Chain mediating effect of regulatory emotional self-efficacy and self-control between self-esteem and aggressiveness in adolescents. Chin. Ment. Health J. 32, 574–579. doi: 10.3969/j.issn.1000-6729.2018.07.007

[ref12] Card N. A. Sawalani G. M. Little S. T. D. (2008). Direct and indirect aggression during childhood and adolescence: a meta-analytic review of gender differences, intercorrelations, and relations to maladjustment. Child Dev. 79, 1185–1229. doi: 10.1111/j.1467-8624.2008.01184.x, PMID: 18826521

[ref13] Chen L. (2010). Study on the correlation among romantic jealousy, personality and their jealousy behaviors. Psychology Exploration. 30, 91–96. doi: 10.3969/j.issn.1003-5184.2010.04.018

[ref14] Chin K. Atkinson B. E. Raheb H. Harris E. Vernon P. A. (2016). The dark side of romantic jealousy. Personal. Individ. Differ. 115, 23–29. doi: 10.1016/j.paid.2016.10.003

[ref15] Crusius J. Mussweiler T. (2012). When people want what others have: the impulsive side of envious desire. Emotion 12, 142–153. doi: 10.1037/a0023523, PMID: 21604867

[ref16] Denson T. F. DeWall C. N. Finkel E. J. (2012). Self-control and aggression. Curr. Dir. Psychol. Sci. 21, 20–25. doi: 10.1177/0963721411429451

[ref17] Desteno D. Valdesolo P. Bartlett M. Y. (2006). Jealousy and the threatened self: getting to the heart of the green-eyed monster. J. Pers. Soc. Psychol. 91, 626–641. doi: 10.1037/0022-3514.91.4.626, PMID: 17014289

[ref18] Desteno D. A. Salovey P. (1996). Evolutionary origins of sex differences in jealousy? Questioning the “fitness” of the model. Psychol. Sci. 7, 367–372. doi: 10.1111/j.1467-9280.1996.tb00391.x

[ref19] Dewall C. N. Baumeister R. F. Chester D. S. Bushman B. J. (2016). How often does currently felt emotion predict social behavior and judgment? A Meta-analytic test of two theories. Emot. Rev. 8, 136–143. doi: 10.1177/1754073915572690

[ref20] Dewall C. N. Baumeister R. F. Stillman T. F. Gailliot M. T. (2007). Violence restrained: effects of self-regulation and its depletion on aggression. J. Exp. Soc. Psychol. 43, 62–76. doi: 10.1016/j.jesp.2005.12.005

[ref21] Finkenauer C. Engels R. Baumeister R. F. (2005). Parenting behaviour and adolescent behavioural and emotional problems: the role of self-control. Int. J. Behav. Dev. 29, 58–69. doi: 10.1080/01650250444000333

[ref22] Fredrickson B. L. (2004). The role of positive emotions in positive psychology - the broaden-and-build theory of positive emotions. Am. Psychol. 359, 1367–1378. doi: 10.1098/rstb.2004.1512, PMID: 11315248 PMC3122271

[ref23] Gao L. Li S. Li G. Yang J. Wang X. (2022). The effect of Adolescents' friendship jealousy on aggression: the chain mediating role of self-esteem and self-control. Chinese. J. Clin. Psychol. 30, 425–428+433. doi: 10.16128/j.cnki.1005-3611.2022.02.035

[ref24] Goldenberg J. L. Landau M. J. Pyszczynski T. Cox C. R. Greenberg J. Solomon S.. (2003). Gender-typical responses to sexual and emotional infidelity as a function of mortality salience induced self-esteem striving. Personal. Soc. Psychol. Bull. 29, 1585–1595. doi: 10.1177/0146167203256880, PMID: 15018688

[ref25] González N. J. Bolaños J. A. C. Ruiz R. O. (2017). Proactive and reactive aggressive behavior in bullying: the role of values. International Journal of Educational Psychology: IJEP. 6, 1–24. doi: 10.17583/IJEP.2017.2515

[ref26] Gu X. Yao R. Li H. Li K. (2012). Path analysis of aggressive behavior and their influencing factors among middle school students. Chin. J. School Health 33, 155–157. doi: 10.16835/j.cnki.1000-9817.2012.02.011

[ref27] Hamza A. Sharma M. K. Marimuthu P. Murli S. (2019). Cognitive behavioral skill-based training program for enhancing anger control among youth. Ind. Psychiatry J. 28, 37–43. doi: 10.4103/ipj.ipj_28_17, PMID: 31879445 PMC6929232

[ref28] He C. Xia M. Jiang G. Wei H. (2012). Mediation role of self-control between internet game addiction and self-esteem. Chin. J. Clin. Psych. 20, 58–60. doi: 10.16128/j.cnki.1005-3611.2012.01.011

[ref29] He T. Zhang J. (2012). Correlation analysis between internal control jealousy and self-esteem in college students. Chin. J. School Health 33, 1248–1250. doi: 10.16835/j.cnki.1000-9817.2012.10.039

[ref30] Hill S. E. Delpriore D. J. Vaughan P. W. (2011). The cognitive consequences of envy: attention, memory, and self-regulatory depletion. J. Pers. Soc. Psychol. 101, 653–666. doi: 10.1037/a0023904, PMID: 21639650

[ref31] Hu F. Chen G. Cai T. (2012). Preliminary study on self-control scale on Chinese middle school students. Chin. J. Health Psychol. 20, 1183–1184. doi: 10.13342/j.cnki.cjhp.2012.08.007

[ref32] Hu Z. (2009). A study on implicit aggressiveness and implicit self-esteem of cyber behavior anomie. Psychol. Sci. 32, 210–212. doi: 10.16719/j.cnki.1671-6981.2009.01.050

[ref33] Huang M. Shi Z. Liu M. (2013). Juvenile Criminals' self-esteem and Aggresiveness: Mediaror effect of self-control. Chin. J. Clin. Psych. 21, 602–604. doi: 10.16128/j.cnki.1005-3611.2013.04.003

[ref34] Lennarz H. K. Lichtvvarck-Aschoff A. Finkenauer C. Granic I. (2017). Jealousy in adolescents' daily lives: how does it relate to interpersonal context and well-being? J. Adolesc. 54, 18–31. doi: 10.1016/j.adolescence.2016.09.008, PMID: 27863267

[ref35] Li D. Wang D. Li D. (2019). Aggressive behavior of Macao middle school students and its influencing factors. Chin. J. School Health 40, 1682–1685. doi: 10.16835/j.cnki.1000-9817.2019.11.023

[ref36] Li H. Yang Z. (2008). Evolutionary theories and empirical studies on gender difference in jealousy. Psychol. Sci. 4, 966–970. doi: 10.16719/j.cnki.1671-6981.2008.04.039

[ref37] Li X. Li Z. Zhang L. (2017). Relationships between social support and aggression of adolescents:the chain mediating roles of self-esteem and self-control. Psychol. Dev. Educ. 33, 240–248. doi: 10.16187/j.cnki.issn1001-4918.2017.02.13

[ref38] Lin C. Yang Z. Huang X. (2003). A dictionary of psychology. Shanghai: Shanghai Education Press.

[ref39] Liu H. Dou K. Yu C. Nie Y. Zheng X. (2021). The relationship between peer attachment and aggressive behavior among Chinese adolescents: the mediating effect of regulatory emotional self-efficacy. Int. J. Environ. Res. Public Health 18:7123. doi: 10.3390/IJERPH18137123, PMID: 34281060 PMC8297157

[ref40] Liu J. Zhou Y. Gu W. (2009). Reliability and validity of Chinese version of Buss-Perry aggression questionnaire in adolescents. Chin. J. Clin. Psych. 17, 449–451.

[ref41] Liu S. Zeng N. Sun Z. Hu M. (2016). Socioecological risk factors among interpersonal violence perpetrators. Chinese Journal of Behavioral Medicine and Brain Science 25, 1051–1056. doi: 10.3760/cma.j.issn.1674-6554.2016.11.020

[ref42] Luo Y. Yu Y. Yang Y. Sun Y. (2011). Correlation study of aggressive behavior and explicit self-esteem among middle school students. Chinese. J. Sch. Health 32, 914–915+918. doi: 10.16835/j.cnki.1000-9817.2011.08.008

[ref43] Murphy A. M. Russell G. (2018). Rejection sensitivity, jealousy, and the relationship to interpersonal aggression. J. Interpers. Violence 33, 2118–2129. doi: 10.1177/0886260515622572, PMID: 26802043

[ref44] Ning J. (2021). Main data of the seventh National Population Census. Chinese Statistics. 5, 4–5. doi: 10.38309/n.cnki.nzgxx.2021.000481

[ref45] Pan Y. (2015). Development of young Adolescents' self-esteem and influencing factors: a longitudinal analysis. Acta Psychol. Sin. 47, 787–796. doi: 10.3724/SP.J.1041.2015.00787

[ref46] Pechorro P. Marsee M. DeLisi M. Maroco J. (2021). Self-control and aggression versatility: moderating effects in the prediction of delinquency and conduct disorder among youth. J. Forensic Psychiatry Psychol. 32, 949–966. doi: 10.1080/14789949.2021.1959627

[ref47] Ren P. Song Z. Meng X. Qin X. Zhang Y. (2021). The relationship between academic achievement, popularity and aggressive behavior of adolescents: a cross-lagged analysis. Psychol. Dev. Educ. 37, 710–718. doi: 10.16187/j.cnki.issn1001-4918.2021.05.12

[ref48] Rentzsch K. Schroder-Abe M. Schuetz A. (2015). Envy mediates the relation between low academic self-esteem and hostile tendencies. J. Res. Pers. 58, 143–153. doi: 10.1016/j.jrp.2015.08.001

[ref49] Robert F. V. Zullig K. J. Asa A. Revels (2017). Aggressive and violent behavior and emotional self-efficacy: is there a relationship for adolescents? J. Sch. Health 87, 269–277. doi: 10.1111/josh.12493 28260243

[ref50] Si J. (2009). The relationship between implicit and explicit self-esteem and jealous behavior in college students. Chin. J. Clin. Psych. 17:3.

[ref51] Sun H. Yu Y. Wang C. Ji L. (2013). The relationship bewteen sel-festeem and aggression in adolescents:the intermediary role of jealousy. Psychology and Innovation Ability Improvement-The 16th National Psychology 1

[ref52] Tan S. Guo Y. (2008). Revision of self-control scale for Chinese college students. Chin. J. Clin. Psych. 16, 468–470.

[ref53] Thompson R. A. (1991). Emotional regulation and emotional development. Educ. Psychol. Rev. 3, 269–307. doi: 10.1007/BF01319934

[ref54] Tian L. (2006). Shortcoming and merits of Chinese version of Rosenberg(1965) self-esteem scale. Psychol. Explor. 2, 88–91. doi: 10.3969/j.issn.1003-5184.2006.02.020

[ref55] Toothaker L. E. (1994). Multiple regression: testing and interpreting interactions. J. Oper. Res. Soc. 43, 453–120. doi: 10.2307/2348581

[ref56] Turner K. A. White B. A. (2015). Contingent on contingencies: connections between anger rumination, self-esteem, and aggression. Personal. Individ. Differ. 82, 199–202. doi: 10.1016/j.paid.2015.03.023

[ref57] Wang X. (2001). A study of the reliability and validity of seven jealousy assessment scales. Psychol. Sci. 5, 573–575+639. doi: 10.16719/j.cnki.1671-6981.2001.05.016

[ref58] Wen Z. Ye B. (2014). Analyses of mediating effects: the development of methods and models. Adv. Psychol. Sci. 22, 731–745. doi: 10.3724/SP.J.1042.2014.00731

[ref59] Xiong H. Zhang J. Ye B. Zheng X. (2012). Common method variance effects and the models of statistical approaches for controlling it. Adv. Psychol. Sci. 20, 757–769. doi: 10.3724/SP.J.1042.2012.00757

[ref60] Yang Z. Luo Y. Gu R. Liu Y. Cai H. (2017). Self-esteem and brain: a social neuroscience approach. Adv. Psychol. Sci. 25, 788–798. doi: 10.3724/SP.J.1042.2017.00788

[ref61] Yu B. Le G. Liu H. (2013). The strength model of self-control. Adv. Psychol. Sci. 21, 1272–1282. doi: 10.3724/SP.J.1042.2013.01272

[ref62] Zhang J. Wang H. Li W. (2006). Research on the jealousy of college students and its influencing factors. Chin. J. School Health 4, 320–321. doi: 10.3969/j.issn.1000-9817.2006.04.027

